# Leukocyte telomere length and hippocampus volume: a meta-analysis

**DOI:** 10.12688/f1000research.7198.1

**Published:** 2015-10-15

**Authors:** Gustav Nilsonne, Sandra Tamm, Kristoffer N. T. Månsson, Torbjörn Åkerstedt, Mats Lekander

**Affiliations:** 1 Stress Research Institute, Stockholm University, Stockholm, Sweden; 2 Department of Clinical Neuroscience, Karolinska Institutet, Stockholm, Sweden; 3 Department of Behavioural Sciences and Learning, Linköping University, Linköping, Sweden; 4 PRIMA Psychiatry, Stockholm, Sweden

**Keywords:** Telomeres, Morphometry, Hippocampus, Microglia

## Abstract

Leukocyte telomere length has been shown to correlate to hippocampus volume, but effect estimates differ in magnitude and are not uniformly positive. This study aimed primarily to investigate the relationship between leukocyte telomere length and hippocampus gray matter volume by meta-analysis and secondarily to investigate possible effect moderators. Five studies were included with a total of 2107 participants, of which 1960 were contributed by one single influential study. A random-effects meta-analysis estimated the effect to *r* = 0.12 [95% CI -0.13, 0.37] in the presence of heterogeneity and a subjectively estimated moderate to high risk of bias. There was no evidence that apolipoprotein E (APOE) genotype was an effect moderator, nor that the ratio of leukocyte telomerase activity to telomere length was a better predictor than leukocyte telomere length for hippocampus volume. This meta-analysis, while not proving a positive relationship, also is not able to disprove the earlier finding of a positive correlation in the one large study included in analyses. We propose that a relationship between leukocyte telomere length and hippocamus volume may be mediated by transmigrating monocytes which differentiate into microglia in the brain parenchyma.

## Introduction

### Background

Telomeres are protective DNA-protein complexes at the ends of eukaryotic chromosomes ^1^. In cells with limited proliferative capacity, telomeres shorten with each cell division ^2^. Thus, telomere length in non-stem cells is a marker for biological aging. Germ cells, stem cells, and some cancer cells are able to maintain telomere length by expressing the enzyme telomerase reverse transcriptase ^1, 2^. Shorter leukocyte telomere length has been linked to adverse health outcomes including cancer ^3^, cardiovascular disease ^4^, and psychiatric disorders ^5^, as well as poor self-rated health outcomes such as sleep quality and daytime functioning ^6^. Recently, the association between leukocyte telomere length and brain morphology has been investigated by several research groups. The hippocampus has been a particular area of interest, possibly because it is a particularly plastic brain region, capable of neurogenesis ^7, 8^ and volume increases e.g. in spatial learning ^9, 10^, but also especially afflicted by atrophy in Alzheimer’s disease ^11^. The relationship between leukocyte telomere length and hippocampus volume has however not been fully clarified by investigations to date.

Two possible moderators of a relationship between leukocyte telomere length and hippocampus volume have been proposed. Wikgren *et al.* ^12^ proposed apolipoprotein E (APOE) genotype as a moderator, while Jacobs *et al.* ^13^ proposed telomerase activity as a moderator, suggesting that the ratio of telomerase activity to telomere length is a better predictor than either measure alone.

### Aims

This meta-analysis aimed primarily to estimate the correlation between leukocyte telomere length and hippocampus gray matter volume. Secondary aims were to investigate the possible moderating effect of APOE genotype on this correlation and to investigate whether the ratio of leukocyte telomerase to telomere length is a better predictor of hippocampus volume than either measure alone. We discuss possible mechanisms for the putative association between leukocyte telomere length and hippocampus volume and point to avenues for further inquiry.

## Methods

**Figure 1.**
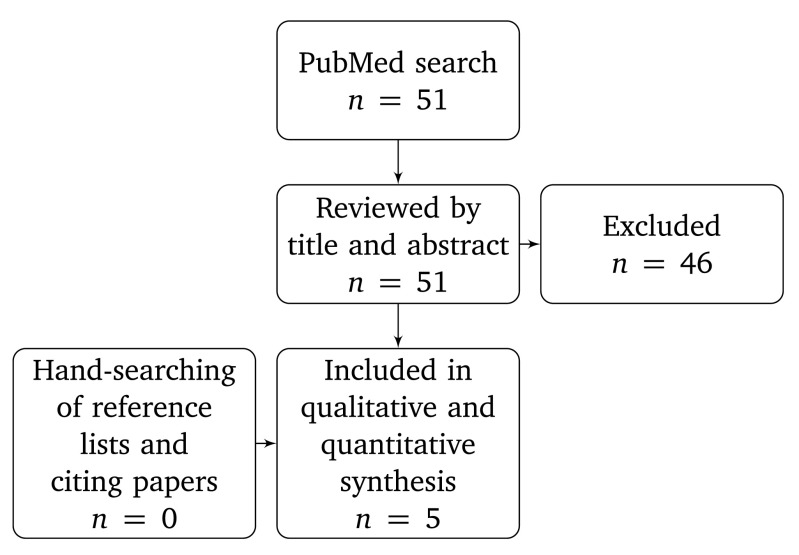
Data inclusion flowchart.

PubMed ( www.pubmed.gov) was searched on 2015-09-22 using the search string ”telomer* hippocampus” with no limitations by date nor language. Results were reviewed by title and abstract. Studies reporting data on the association between leukocyte telomere length and hippocampus volume in humans were included. Search results were reviewed independently by one investigator (GN) and by a committee of three investigators (KM, TÅ, and ML), reaching the same sample selection. Data were extracted by one investigator (GN) and checked for accuracy by another investigator (ST). The following variables were coded: number of participants and their sex distribution and age, observed correlation between leukocyte telomere length and hippocampus volume, analysis covariates, and whether apolipoprotein E (APOE) genotype or telomerase activity were also analysed. Figure 1 shows data inclusion. We analysed data with R version 3.2.0 ^14^ with the metafor ^15^, psych ^16^, and pwr ^17^ packages. A random-effects model was fitted due to heterogeneity between studies. A sensitivity analysis investigating the effect of excluding one study was performed post-hoc. The meta-analysis protocol was not predefined, and hence not registered. To compare telomere length, telomerase activity, and their ratio, data were estimated from published scatterplots using the freely available WebPlotDigitizer ( http://arohatgi.info/WebPlotDigitizer/) version 3.8 ^18^.

One study (Jacobs *et al.* ^13^) reported results for right and left hippocampus separately. Partial *r* ^2^ values for the relationship between hippocampus volume and leukocyte telomere length were however identical for both sides ( *r* ^2^ = 0.16) and this value was used. The same study reported data only for one subset of participants (APOE *∈*4 non-carriers). All code and data needed to reproduce the analyses in this paper are available at 19, as is the list of PubMed search results.

## Results

### Included studies

Five studies were included, of which two each contained two different participant cohorts, with a total of 2107 participants ( Table 1). Several possible sources of heterogeneity were identified in sampling strategies and analysis methods. Grodstein *et al.* ^20^ included a sub-group of patients with minimal cognitive impairment, and Wolkowitz *et al.* ^21^ included a cohort with major depressive disorder. All studies determined relative average telomere length by PCR as the ratio of telomeres to single genes (T/S ratio). Two studies (Grodstein *et al.* ^20^ and King *et al.* ^22^) used the T/S ratio in statistical analyses, whereas the other three studies (Wikgren *et al.* ^12^, Jacobs *et al.* ^13^, and Wolkowitz *et al.* ^21^) determined the average telomere base pair length and used that for statistical analyses. One study (King *et al.* ^22^) log-transformed telomere length data prior to analysis in order to better approximate a normal distribution. Three studies (Grodstein *et al.* ^20^, Jacobs *et al.* ^13^, and Wikgren *et al.* ^12^) did not use a log-transformation, and one study (Wolkowitz *et al.* ^21^) reported that some variables were log-transformed but did not say whether telomere length was one of them.

**Table 1.** Characteristics of included studies. APOE = Apolipoprotein E genotype, investigated (+) or not investigated (-); TA = telomerase activity, investigated (+) or not investigated (-); *∈*3 & *∈*4 = APOE alleles; MDD = Major Depressive Disorder; ^a^ = Age was not given for imaging subsample, age for whole healthy subsample substituted; ^b^ = Median and interquartile range.

Study and group	Year	Ref.	*n*	Female, *n*	Age, mean (SD)	Covariates	APOE	TA
Grodstein	2008	20	26	26 (100%)	79.2 (2.2) ^a^	age, education	-	-
Wikgren *∈*3/ *∈*3	2012	12	29	18 (62%)	62.1 (8.5)	age, body size	+	-
Wikgren *∈*4	2012	12	28	18 (64%)	61.1 (8.5)	age, body size	+	-
King	2014	22	1960	1153 (59%)	50 (42 – 58) ^b^	age, sex, race/ethnicity	+	-
Wolkowitz MDD	2015	21	19	12 (63%)	37.8 (12.0)	age, sex	-	+
Wolkowitz control	2015	21	17	11 (59%)	34.9 (9.6)	age, sex	-	+
Jacobs non- *∈*4	2015	13	28	28 (100%)	58.0 (4.7)	age, education, BMI	+	+

Hippocampus volume was determined using magnetic resonance imaging in all included studies. King *et al.* ^22^, Jacobs *et al.* ^13^, and Wolkowitz *et al.* ^21^ used FreeSurfer ( http://freesurfer.net/) for automated parcellation. Wikgren *et al.* ^12^ used manual tracing and did not specify whether the person performing the tracing was blind to other participant outcomes at the time. Grodstein *et al.* ^20^ did not specify how hippocampus volumes were determined. Covariates for analyses differed between studies ( Table 1). No study fractionated leukocytes nor analysed the relative contributions of different leukocyte subsets.

### Correlation of leukocyte telomere length to hippocampus volume, quantitative synthesis

**Figure 2.**
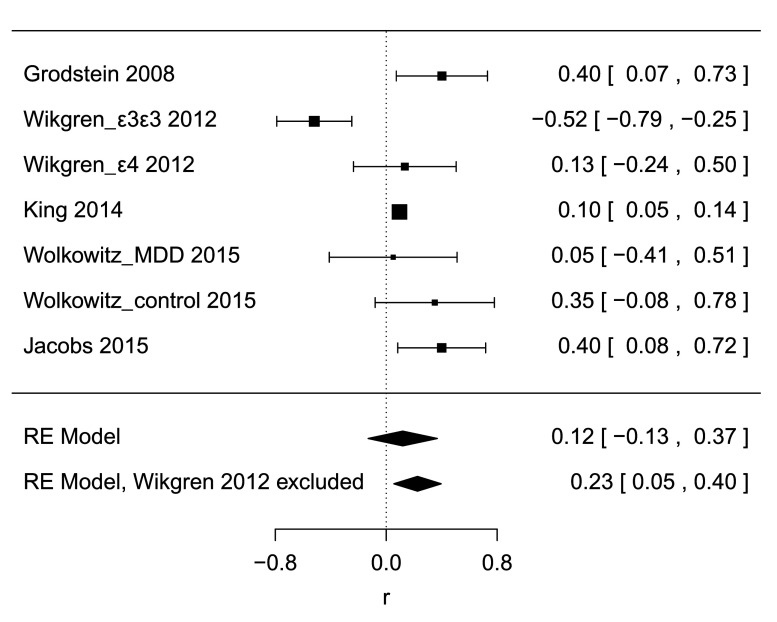
Forest plot. Diamonds at bottom show estimates for a random-effects model including all studies and an estimate when excluding one study as a sensitivity analysis.

A random-effects meta-analysis of all 7 datasets yielded a summary estimate of *r* = 0.12 [95% CI -0.13, 0.37], *p* = 0.35, Figure 2, indicating a positive direction of the relationship between telomere length and hippocampus volume, but failing to show a significant difference from 0. A test for heterogeneity was significant ( *τ* ^2^ = 0.086, *I* ^2^ = 0.84, *H* ^2^ = 6.25, *Q*_*df*=6_ = 28.1, *p* < 0.0001), the Q-Q plot indicated deviation from normality, and the trim-and fill-method to test sensitivity for publication bias imputed two additional studies and yielded an estimate of *r* = 0.01 [95% CI -0.23, 0.25], *p* = 0.96, calling into question whether model assumptions were satisfied and suggesting that the effect estimate may be an overestimation due to publication bias. In spite of the small number of studies, we explored meta-regression as a means to address study heterogeneity, using mean age and fraction of female participants as independent variables. Neither was significantly associated (age: *r* ^2^ = 0.00, *p* = 1.00; sex: *r* ^2^ = 0.23, *p* = 0.14).

**Figure 3.**
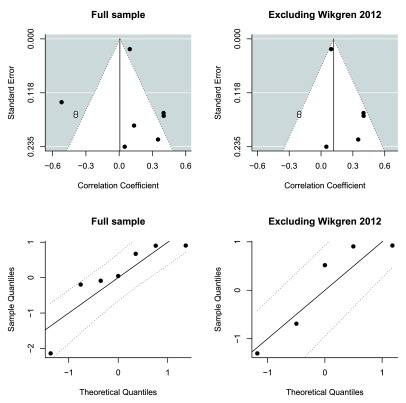
Funnel plots and Q-Q plots. Funnel plots show that the sample was right-skewed. Trim-and-fill analysis imputed two additional studies (unfilled circles) both in the full sample and in the reduced sensitivity analysis sample. Q-Q plots show that in the full sample, there was deviation from linearity due to one study. When this study was removed in the sensitivity analysis, linearity was acceptable.

The observed deviation from normality was mostly due to the *∈*4/ *∈*4-positive cohort from Wikgren 2012 ^12^ ( Figure 3). In order to investigate the sensitivity of the meta-analysis to this particular study, the analysis was performed again without it. The meta-analytic estimate was now *r* = 0.23 [95% CI 0.05, 0.40], *p* = 0.01, without significant heterogeneity, although power to detect heterogeneity was now very limited ( *τ* ^2^ = 0.017, *I* ^2^ = 0.48, *H* ^2^ = 1.93, *Q*_*df*=4_ = 7.9, *p* = 0.09), and with adequate normality as judged by inspection of the Q-Q plot ( Figure 3). The trim-and fill-method to test sensitivity for publication bias imputed two additional studies and yielded an effect estimate of *r* = 0.12 [95% CI -0.07, 0.31], *p* = 0.22 ( Figure 3).

Risk of bias was assessed qualitatively and by inspection of funnel plots and Q-Q plots. Two of the included studies reported results only for subsets of participants, without indicating that these sub-group analyses were specified on beforehand. Grodstein *et al.* ^20^ reported results only for healthy participants and participants with minimal cognitive impairment (MCI), leaving out 5 participants with dementia. Jacobs *et al.* ^13^ reported results only for APOE *∈*4 non-carriers, leaving out 19 APOE *∈*4 carriers. Only one study (Wolkowitz *et al.* ^21^) showed negative results. None of the studies reported blinding of experimenters carrying out laboratory nor statistical analyses. All studies except King *et al.* ^22^ reported fewer than 30 participants. With 30 participants and using the effect estimate of *r* = 0.12 found in the main analysis, power would be 0.10. The largest effect estimate in this paper, observed in the sensitivity analysis, was *r* = 0.23, for which 30 participants would yield a power of 0.23. The observed low power among the included studies is not remarkable compared to the neuroscience field in general ^23^. In combination with the preponderance of observed significant associations, it gives rise to a suspicion of bias. Funnel plots showed that the sample was right-skewed, although the small number of studies limits interpretation. The risk of bias was subjectively estimated to be moderate to high.

### Correlation of leukocyte telomere length to hippocampus volume, qualitative synthesis

A qualitative synthesis takes as point of departure the large study by King *et al.* ^22^, since it contributes most of the participants (1960 of 2107) in this meta-analysis. This study was based on the Dallas Heart Study 2 cohort, a sample of 3401 participants examined in 2007–2009 and intended to be representative of the adult population of Dallas County, Texas. 2082 of these participants underwent magnetic resonance imaging and the final sample contained 1960 individuals. 48 brain regions were investigated using FreeSurfer. A positive direction of the association between leukocyte telomere length and brain volume was found for all 46 parenchymatous brain regions, whereas a negative direction of the association was found for the lateral and inferior lateral ventricles. Associations were significant at *α* = 0.05 in 27 regions after correcting for multiple comparisons using the false discovery rate method. The strongest association was found for the precuneus ( *r* = 0.13), followed by the thalamus ( *r* = 0.11), and then the hippocampus, lateral orbitofrontal cortex, inferior parietal cortex, posterior parietal cortex, inferior temporal cortex, and posterior cingulate cortex (all *r* = 0.10). Leukocyte telomere length predicted both total cerebral volume ( *r* = 0.12) and total cortical volume ( *r* = 0.11). The relationship between leukocyte telomere length and hippocampus volume was moderated by age and was strongest in older participants. Authors reported that only marginal effects were seen when adjusting for hypertension, obesity, diabetes mellitus, and smoking status. The final model adjusted for age, sex, and ethnicity. Hippocampal and cortical volumes were found in an overlapping sample to correlate to cognitive performance using the Montreal Cognitive Assessment ^24^, supporting the validity of these volumetric measurements.

Demographic and clinical variables associated to leukocyte telomere length in the Dallas Heart Study 2 cohort have been reported in a previous paper including 3155 participants ^25^. With telomere length categorized into tertiles, there was a negative correlation to monocyte fraction (short tertile: 6.88% (SD 2.21), middle tertile: 6.75% (SD 2.10), long tertile: 6.53% (SD 2.03), *p _adj_* = 0.03), but not to other leukocyte fractions. Leukocyte telomere length also correlated positively to education and income, even when adjusting for age, gender and ethnicity. Since education and income were not analysed in King *et al.* ^22^, they may represent unknown confounders or effect mediators.

In summary, the association between leukocyte telomere length and hippocampus volume found by King *et al.* ^22^ gains credence from the large sample and the population-based sampling strategy. The consistent association found across parenchymatous brain regions further supports the finding of an effect in the hippocampus. The previously reported finding that leukocyte subsets were mostly stable across telomere length tertiles in the Dallas Heart Study 2 is reassuring, in that it suggests that apparent differences in telomere length were not due to differences in cell type composition, although such confounding cannot be fully ruled out by this analysis.

The other included studies besides King *et al.* ^22^ all had samples for which results could be estimated of *n* < 30. While samples were small, reported effects were in the positive direction with the exception of one sub-group (from Wikgren *et al.* ^12^), and estimated effects were larger than in King *et al.* ^22^ with the exception of one further sub-group (from Wolkowitz *et al.* ^21^). This pattern of results is expected in the presence of bias.

Qualitatively, the overall pattern of results favors a positive association between leukocyte telomere length and hippocampus volume.

### Effect of APOE genotype

The effect of APOE genotype on the relationship between telomere length and hippocampus volume, first proposed by Wikgren *et al.* ^12^, has been subsequently investigated by King *et al.* ^22^ and by Jacobs *et al.* ^13^. Wikgren *et al.* ^12^ reported a different effect magnitude in two groups ( *∈*3/ *∈*3 homozygotes: *r* = -0.52; *∈*4 carriers: *r* = 0.13), but not an interaction test which would tell whether that difference was statistically significant. In contrast, Jacobs *et al.* ^13^ reported a positive association ( *r* = 0.40) only among *∈*4-noncarriers. Jacobs *et al.* ^13^ also did not test whether the difference between groups was statistically significant. King *et al.* ^22^ reported no significant effect of APOE *∈*2, *∈*3 nor *∈*4 genotype in 1409 participants for whom APOE data were available. It was not possible to investigate these results quantitatively as effect sizes were not reported. In summary, none of the included studies provide positive evidence for a moderating effect of APOE genotype on the association between telomere length and hippocampus volume.

### Leukocyte telomerase/telomere length ratio as predictor

Two studies with four datasets investigated leukocyte telomerase activity in addition to leukocyte telomere length as predictors for hippocampus volume. Jacobs *et al.* ^13^ proposed that the leukocyte telomerase/telomere length ratio is a better predictor for hippocampus volume than either measure alone, but without formally testing whether the ratio yielded a higher correlation. We decided to investigate this proposition. Since it was necessary to perform a test for dependent correlations, and the correlations between telomere length and telomerase activity were not given, we extracted data from scatterplots presented in both papers and calculated the required correlations from the estimated data. Comparisons by inspection of original plots and plots of estimated data showed satisfactory data extraction accuracy and can be viewed at 19. Pairwise estimates of hippocampus volume correlated well ( *r*’s = 0.99-1.00), further supporting successful data extraction.

In Jacobs *et al.* ^13^, telomere length and telomerase activity did not correlate (left: *r* = -0.19 [95% CI -0.19, 0.53], *p* = 0.32; right: *r* = -0.14 [95% CI -0.49, 0.25], *p* = 0.48; Note that while these measures have no intrinsic laterality, data were extracted separately for the right and left sides, and the difference between sides is likely due to estimation error). The ratio of telomerase activity to telomere length was a better predictor for hippocampus volume than telomerase activity alone for the right side but not the left. On neither side was the ratio a better predictor than telomere length ( Table 2). In Wolkowitz *et al.* ^21^, telomere length and telomerase activity did not correlate (healthy controls: *r* = 0.09 [95% CI -0.44, 0.58], *p* = 0.75; patients with major depressive disorder: *r* = 0.14 [95% CI -0.34, 0.56], *p* = 0.57). The ratio of telomerase activity to telomere length was not a better predictor than telomerase activity nor telomere length in either group ( Table 2). It is notable that correlations between telomerase activity and hippocampus volume differed in direction between the two studies ( Table 2).

**Table 2.** Comparisons of the ratio of telomerase to telomere length as predictor of hippocampus volume to either measure alone. Data from Jacobs *et al.* ^13^ were estimated from published scatterplots showing only the APOE *∈*4-negative participant subset, separately for right and left hippocampus, and are adjusted for age. Further adjustments for education and BMI were not possible because data were not available. Data from Wolkowitz *et al.* ^21^ were estimated from published scatterplots and are not adjusted for any covariates. Adjustments for age and sex were not possible because data were not available. TA = telomerase activity; TL = telomere length; HV = hippocampus volume; Ratio = ratio of telomere activity to telomere length; MDD = major depressive disorder; ^a^ = effect direction favors telomere length or activity over ratio.

Study and group	*n*	TL and HV *r* [95% CI]	TA and HV *r* [95% CI]	Ratio and HV *r* [95% CI]	Ratio vs TA *p*	Ratio vs TL *p*
Jacobs left side	28	0.48 [0.13, 0.72]	-0.33 [-0.62, 0.05]	-0.37 [-0.65, -0.00]	0.17	0.59 ^a^
Jacobs right side	28	0.56 [0.23, 0.77]	-0.28 [-0.59, 0.11]	-0.35 [-0.64, 0.03]	0.005	0.30 ^a^
Wolkowitz MDD	19	0.07 [-0.40, 0.51]	0.58 [0.17, 0.82]	-0.41 [-0.73, 0.05]	0.49 ^a^	0.27
Wolkowitz control	16	0.09 [-0.44, 0.57]	0.18 [-0.36, 0.64]	-0.00 [-0.51, 0.51]	0.56 ^a^	0.81 ^a^

## Discussion

### Main findings and interpretation

We investigated the correlation between leukocyte telomere length and hippocampus volume. In the main analysis, we found a correlation of *r* = 0.12 with considerable uncertainty and not significantly different from 0. Studies were heterogeneous and the exclusion, as a sensitivity analysis, of one study (Wikgren *et al.* ^12^) shifted the estimate to *r* = 0.23, which was significantly different from 0. Trim-and-fill analyses to investigate publication bias yielded imputations which brought the estimated effect down to *r* = 0.01 in the full sample and *r* = 0.12 in the sample excluding one study, neither of which was significantly different from 0.

A meta-analysis does not necessarily yield better estimates than a single high-powered study, mainly because a high-powered study can be expected to have low bias, and if smaller studies with more bias are included in a meta-analysis, then the resulting estimate may be more biased and hence less accurate than that of the single study ^26^. Therefore, while this meta-analysis failed to corroborate a positive association, we argue that it does not provide compelling evidence to reject the positive estimate from the large study by King *et al.* ^22^, which contributed 1960 out of 2107 participants.

We found no evidence for APOE genotype as an effect moderator between leukocyte telomere length and hippocampus volume. The ratio of telomerase activity to telomere length was a better predictor than telomerase activity on one side in Jacobs *et al.* ^13^ but not in either subsample in Wolkowitz *et al.* ^21^. We conclude that there is no evidence that APOE genotype moderates the relationship between leukocyte telomere length and hippocampus volume, while the evidence that the telomerase activity to telomere length ratio predicts hippocampus volume better than telomerase activity is inconclusive.

### Limitations

An important limitation of this meta-analysis is that none of the included studies fractionated leukocytes before analysis, meaning that any change in the leukocyte cell type composition may confound results. Hippocampal atrophy has been linked to outcomes that are also associated to changes in leukocyte subset composition, such as inflammation and sleep pattern changes ^27, 28^. Therefore, leukocyte cell type composition is a possibly important and uninvestigated confounder.

Furthermore, this meta-analysis was limited by the small number of included studies, and by the relatively small sample sizes in all studies except one. Studies were heterogeneous and the risk of bias was subjectively judged to be moderate to high. Since none of the studies published their data in a format conducive to easy reuse, some analyses were precluded. Estimation of data from published scatterplots gives rise to measurement error, which, while probably small, could affect the results. Participant samples were predominantly middle-aged and all included more women than men, limiting external validity.

### Potential mechanisms linking leukocyte telomere length to hippocampus volume

**Figure 4.**
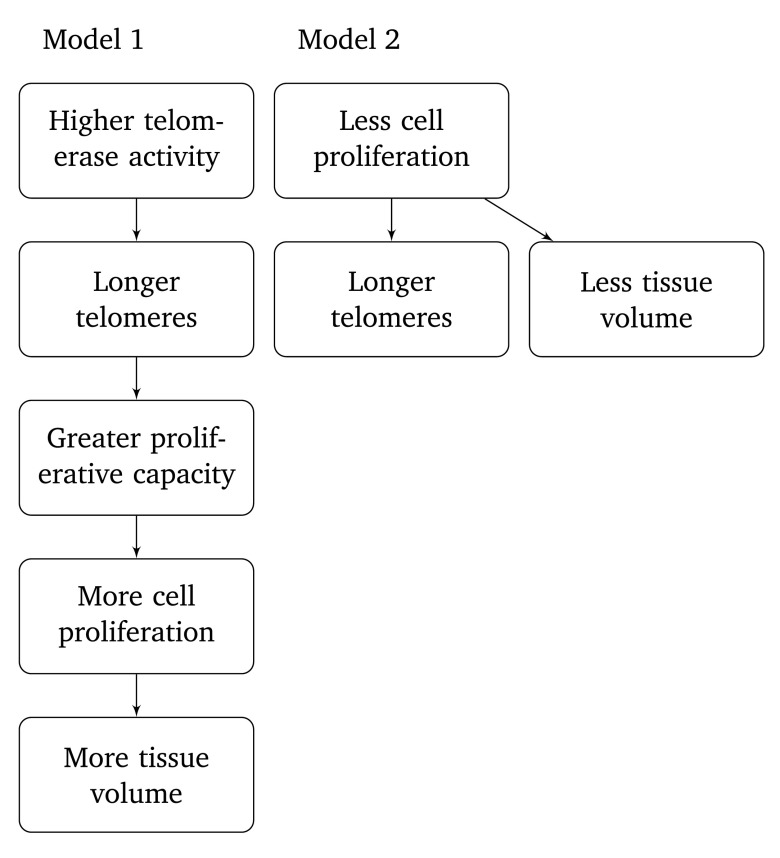
Two models proposed in earlier literature for relationships between telomere length and hippocampus volume.

Different models have been proposed to explain the relationship between leukocyte telomere length and hippocampus volume. King *et al.* ^22^ have proposed, as one possibility among others, that longer telomeres are a surrogate marker for higher telomerase activity, causing greater proliferative capacity and more cell proliferation ( Figure 4, model 1). Wikgren *et al.* ^12^, on the other hand, have proposed that longer telomeres indicate fewer cell divisions have taken place, consistent with fewer cells and a smaller tissue volume ( Figure 4, model 2). These two models make contradictory predictions, and if one accepts a positive correlation between leukocyte telomere volume and hippocampus volume, then a process according to model 2 cannot constitute a dominating contribution to the observed effect. The positive correlation between leukocyte telomerase activity and telomere length predicted by model 1 was however not demonstrated in the present datasets.

A missing link in models 1 and 2 is the relationship between circulating leukocytes and brain parenchyma, as regards telomere length and cell turnover. Leukocyte telomere length represents an average over a large number of white blood cells of different types ^6, 29^. While peripheral leukocytes are a heterogeneous cell population, they share a histogenetic origin in the hematopoietic stem and progenitor cells of the bone marrow. By contrast, neurons and macroglia (oligodendrocytes, and astrocytes) all derive from neuronal stem cells. Neurogenesis continues to a limited extent in adulthood, especially in the hippocampus and subventricular zone ^7, 8, 30^. Microglia, unlike neurons and macroglia, are derived from circulating mononuclear cells which originate in early fetal life from yolk sac hematopoietic stem cells, and later from mesodermally derived hematopoietic stem cells which in adults locate chiefly in the bone marrow ^47^. Whether these two classes of hematopoietic stem cells have the same or different origin is not known ^31^. There is controversy about the extent to which mononuclear cell transmigration and microglial differentiation continues in adult life. Studies using bone marrow transplantation in mice have found that 0 – 25% of microglia derived from the transplanted bone marrow after 4 – 15 weeks ^32– 38^. These findings have been interpreted as evidence both for and against a substantial contribution of bone marrow-derived cells to the microglial cell population in adulthood. In particular, it is not yet clear to what extent injury to the blood-brain barrier (induced, for example, by radiation) facilitates transmigration, and to what extent bone marrow-derived microglia remain in the brain after the resolution of local inflammation. To date, only one study has investigated the correlation between telomere length in leukocytes and in human postmortem brain tissue, and it found a moderate correlation of *r* = 0.42 in a study including 29 patients, all with Alzheimer’s disease ^39^.

Experiments using knock-out mouse models have shown that monocyte recruitment to the brain depends on monocyte chemoattractant protein-1/chemokine (C-C motif) ligand 2/small inducible cytokine A2 (MCP-1/CCL2) signalling trough the CCR2 receptor and that monocyte recruitment to the brain increases in peripheral inflammation ^40^. MCP-1 knock-out mice had less microglial recruitment to the hippocampus and more neurogenesis following cranial irradiation ^41^. Activated microglia can secrete neurotoxic factors including proinflammatory cytokines and reactive oxygen species (ROS), and microglial activation has been proposed as an important pathophysiological process in Alzheimer’s and Parkinson’s dementias ^27, 42^. Leukocyte telomere length has however not been conclusively linked to Alzheimer’s and Parkinson’s dementias in humans ^43– 45^. Zhou *et al.* ^46^ found that telomerase inhibition caused impaired hippocampal neurogenesis, while overexpression of telomerase reverse transcriptase (TERT) led to increased hippocampal neurogenesis.

**Figure 5.**
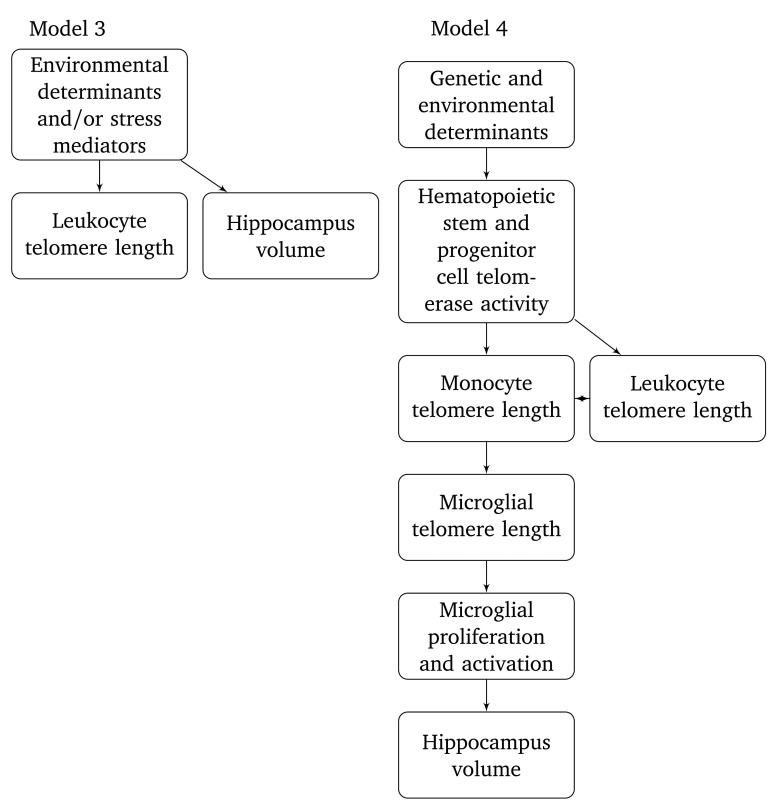
Two models proposed here for relationships between telomere length and hippocampus volume.

It is of course possible that an association between leukocyte telomere length and hippocampus volume could arise from factors determining both outcomes independently ( Figure 5, model 3). King *et al.* ^22^ speculate that chronic inflammation may be an important determinant. Wolkowitz *et al.* ^21^ add that oxidative stress or endogenous hormonal regulation might influence telomerase activity and cell turnover in distant tissues such as leukocytes and brain. Lindqvist *et al.* ^5^ similarly point to inflammation, oxidative stress, and cortisol signalling as possible mechanisms. None of these possible determinants are however clearly supported by empirical data at the present time. Model 3 can be seen as a reformulation of reasoning from these aforementioned papers. Alternatively, we propose speculatively that microglia are a mechanistic link between leukocyte telomere length and hippocampus volume ( Figure 5, model 4). According to this model, hippocampus volume is affected by microglial telomere length by processes related to microglial proliferation and activation. Possibly, longer telomeres could prevent pathological activation of microglia which causes neuronal damage. This model is highly tentative, but it does have the important advantage of providing a possible mechanism by which leukocyte telomere length can affect the cell turnover kinetics of neurons and macroglia, even though they have completely different histogenesis. One observation consistent with the proposed model is that monocytes were the only leukocyte subset correlated to leukocyte telomere length in the Dallas Heart 2 study, even though the effect was small ^25^. More research is needed to verify or falsify testable predictions arising from this model.

## Conclusions

The high-powered study by King *et al.* ^22^ estimated a positive correlation of *r* = 0.10 between leukocyte telomere length and hippocampus volume. The present analysis does not provide compelling evidence against this finding, while it also does not provide absolute evidence in favor. The main meta-analytic effect estimate was *r* = 0.12, with estimates ranging from *r* = 0.00 to *r* = 0.23 after trim-and-fill imputation and in sensitivity analyses. We found no support for a moderating effect of APOE genotype and inconclusive evidence for using the telomerase activity/telomere length ratio as a predictor for hippocampus volume instead of telomere length.

More research in this area is needed to answer several outstanding questions. Differential analyses of white blood cells will be important in order to verify that apparent differences in leukocyte telomere length are not in fact differences in leukocyte cell type composition. Studies comparing telomere lengths in peripheral blood cells and in neurons, macroglia, and microglia will help elucidate the possible mechanistic link due to monocyte-microglial differentiation. Longitudinal designs will allow investigation of whether telomere shortening precedes brain atrophy, or vice versa. Further study of the relationships between leukocyte telomere length, brain volume, and environmental, physiological, and behavioral determinants such as inflammation, sleep and endocrine signalling will help elucidate possible mechanistic factors. Multivariate predictive modelling will be useful to investigate putative diagnostic and/or prognostic properties of telomere length measures. Studies with large samples and open publication of data are greatly to be desired.

## Data availability

ZENODO: Hippocampus-volume-telomere-length: Release for publication, doi: 10.5281/zenodo.31771 ^19^
