# Supplementary material for: Leukocyte telomere length and hippocampus volume: a meta-analysis
Source: F1000Res. 2015 Oct 15;4:1073. [Version 1] doi: 10.12688/f1000research.7198.1 (PMC4670011; doi:10.12688/f1000research.7198.1)
Supplement: Peer review history

# Peer review: Referee response for version 1

**DOI:** 10.5256/f1000research.7754.r10997

**Published:** 2015-11-24

**Authors:** Owen M. Wolkowitz

**Affiliations:** 1 Department of Psychiatry, University of California, San Francisco, San Francisco, CA, USA

This is a well done study with appropriate analyses.

It is timely and of interest.

It is limited (as appropriately acknowledged by authors) by the small number of studies and the large impact of one study (King).

There is a very good discussion of different theoretical models.

It would be worth also highlighting in the abstract their analyses of telomerase itself, not just the TL/telomerase ratio.

They present a secondary analysis without Wikgren. This was a *post-hoc* decision, but this is acknowledged, and the main findings of the meta-analysis include that study.

The only real issue:

- There should have more discussion of the role of moderators (although this information was not available across all studies), e.g.,  age, sex, illness (medical and psychiatric), medications, education, SES, as well as MRI field strength, etc. Indeed, the authors reported that only marginal effects were seen when adjusting for hypertension, obesity, diabetes mellitus, and smoking status. This should be given more emphasis.

I have read this submission. I believe that I have an appropriate level of expertise to confirm that it is of an acceptable scientific standard.

# Peer review: Referee response for version 1

**DOI:** 10.5256/f1000research.7754.r10826

**Published:** 2015-11-02

**Authors:** Kevin S. King

**Affiliations:** 1 Department of Radiology, University of Texas Southwestern Medical Center, Dallas, TX, USA

The current systematic review and meta-analysis of works evaluating the possible link between leukocyte telomere length and hippocampus volume is robust and thorough. It provides a useful quantitative analysis of the current research data and also evaluates potential theories on underlying mechanisms. My reviews are often more lengthy but I think this present work is comprehensive and I really do not think I have a lot to add. 

We were intrigued by existing studies but were worried about potential for bias in existing studies. A particular cause for concern was the small sample size of the studies and relatively modest size of the association being studied. This article gives concrete analysis to support this and determined the power of studies with a sample size of 30 to show an association was only 10%. As mentioned in this rather comprehensive work, some of the work stated that their findings were hypothesis generating and the associations tested were not stipulated at the outset but rather emerged in subgroup analysis. Thanks is still owed to these smaller studies for pioneering the ideas underlying this work. We were rather fortunate to have as a partner the Dallas Heart Study which made our work possible and are grateful for their support.

I have read this submission. I believe that I have an appropriate level of expertise to confirm that it is of an acceptable scientific standard.
